# Trends and Disparities in Secondary Malignant Neoplasms of the Bone in the United States: The WONDER Study

**DOI:** 10.3390/cancers18121877

**Published:** 2026-06-09

**Authors:** Eileen Leach, Andrew Carlson, Abubakar Tauseef, Vikram Murugan

**Affiliations:** School of Medicine, Creighton University, Omaha, NE 68178, USA

**Keywords:** secondary malignancy of the bone, secondary osseous neoplasms, metastatic bone cancer, cancer screening, CDC WONDER

## Abstract

**Background/Objectives**: Secondary malignant neoplasms of the bone are a major concern for cancer patients, with significant morbidity and mortality despite advancements in cancer care. The current literature reports demographic variability, but population-level trends and survivor-specific risk factors remain poorly characterized for bone metastasis. This study aims to provide a comprehensive temporal analysis of the trends and disparities of secondary malignant neoplasms of the bone. **Methods**: Deidentified death certificate data from the United States as reported in the CDC WONDER database (1999–2023) were analyzed for secondary malignant bone neoplasms (ICD-10 C79.5). Age-adjusted mortality rates per 100,000 were calculated and stratified by gender, age, and race. Temporal trends were assessed using Joinpoint Regression, yielding APCs, AAPCs, and 95% CIs. **Results**: Between 1999 and 2023, there were 424,811 deaths in U.S. adults over the age of 25 years related to secondary malignant bone neoplasms. The overall age-adjusted mortality rate (AAMR) increased from 5.77 (95% CI 5.67 to 5.87) in 1999 to 11.92 (95% CI 11.79 to 12.05) in 2023. The average annual percentage change (AAPC) over this period was 3.28 (95% CI 2.63 to 3.94). From 1999 to 2009, mortality decreased with an annual percentage change (APC) in AAMR of −3.62 (95% CI −4.92 to −2.30), followed by a sharp increase in mortality between 2009 and 2023 with an APC of 8.52 (95% CI 7.83 to 9.19). Certain populations had greater increases in AAMR over the study period. The population aged 85 years and older had the largest increase in mortality from secondary malignant bone neoplasms with an AAPC of 4.77 (95% CI 3.38 to 4.77). Over the study period, Black individuals had an overall AAPC of 1.53 (95% CI 0.11 to 2.97), White individuals had an AAPC of 2.60 (95% CI 1.61 to 3.60), and Asian or Pacific Islanders had an AAPC of 3.74 (95% CI 1.17 to 6.38). Rural areas had overall higher AAMRs compared to urban areas, with an AAPC of 2.27 (95% CI 1.52 to 3.02). **Conclusions**: Overall, AAMR increased from 1999 to 2023, though there was a period of decreasing AAMR from 1999 to 2009. We suggest that due to increased rates of screening, it is possible that rates of secondary metastases of the bone have been constant over the years, but they were not diagnosed, leading to a false increase in AAMR. Mortality rates were highest in Black individuals in our study, possibly pointing towards discrepancies in cancer screening and treatment between races. Additionally, the AAMR was higher in rural than in urban areas, which we hypothesize may be due to limited access to treatment, such as surgery. Our study was limited by its nature as a database study, but future studies should focus on the prevention of primary malignancies and minimizing risk factors for the development of secondary malignancies.

## Simple Summary

In this analysis of the CDC WONDER database, we looked at trends in mortality in the United States due to secondary malignant neoplasms of the bone. Age-adjusted mortality rates (AAMRs) were calculated for patients who died of secondary malignant neoplasms of the bone and were then stratified by gender, age, and race. There were 424,811 deaths attributed to secondary malignant neoplasms of the bone between 1999 and 2023. Overall, AAMR increased throughout the study period, though there were greater increases in certain populations, including adults aged 85 years and older, Black patients, and patients in rural areas. This increase in AAMR could be attributed to increased rates of screening as well as more advancements in screening modalities. We hypothesize that higher AAMR in specific racial groups and in rural areas might be due to discrepancies in screening and treatment. Future studies should focus on the prevention of primary malignancies and minimizing risk factors for the development of secondary malignancies.

## 1. Introduction

Secondary malignant neoplasms of the bone are defined as neoplasms that arise from a primary site elsewhere in the body and metastasize to the bone. Bone metastasis is a major source of morbidity among individuals with advanced malignancy, causing intense pain, spinal cord compression, pathologic fractures, and hypercalcemia [1]. Most commonly, breast, prostate, and lung cancers metastasize to the skeleton. Despite advancements in oncologic care, bone metastasis remains a significant marker for poor prognosis and a high burden on the healthcare system [2].

Due to the poor prognosis, early detection and intervention have been at the forefront of research on bone metastasis for multiple decades [3]. Imaging techniques, such as positron emission tomography–computed tomography (PET/CT), single-photon emission computed tomography–computed tomography (SPECT/CT), and whole-body magnetic resonance imaging (MRI) with diffusion-weighted imaging, have allowed for earlier detection and tailored intervention for bone metastasis [4]. Clinically, treatment options are targeted at controlling pain, preventing skeletal-related events (SREs), and limiting systemic progression [5]. Due to a lack of treatment options to significantly improve the prognosis, prevention remains paramount. Therefore, understanding the trends and disparities in who is most affected by bone metastasis is necessary.

Prior studies of the Centers for Disease Control and Prevention Wide-ranging ONline Data for Epidemiologic Research (CDC WONDER) database have focused on the primary malignant neoplasms of the bone or secondary malignancies, but few studies have specifically focused on secondary malignant neoplasms of the bone [6,7]. In the landscape of bone metastasis, there have been several other population-level analyses and registry studies characterizing the distribution of bone involvement by primary site of cancer and associated disparities. Prior research using administrative data and cancer registries has demonstrated that lung cancer, breast cancer, and prostate cancer disproportionately contribute to sex, race/ethnicity, and socioeconomic status disparities observed in patients with bone metastasis [8,9]. These registry-based reports have variable completeness and were introduced at different times across data systems, limiting the comparability of the results.

Vital knowledge gaps remain regarding trends and disparities of bone metastasis. Although most studies have reported the incidence or prevalence of bone metastasis before death, fewer studies have used nationally collected death certificate data to examine the temporal trends and disparities in mortality associated with bone metastasis [9,10,11]. This study is one of the largest to use national death certificate data to quantify and analyze the trends and disparities in secondary malignant neoplasms of the bone.

This study aims to utilize the most current CDC WONDER multiple-cause mortality data (1999–2023) to provide a comprehensive temporal analysis of the trends and disparities of secondary malignant neoplasms of the bone across age, sex, race/ethnicity, state, and urban/rural categories within the United States. Improving our understanding of trends and disparities in secondary neoplasms of the bone will help guide targeted public health interventions for vulnerable populations, oncologic care, and future research.

## 2. Materials and Methods

The CDC WONDER Multiple Cause of Death database was queried for deaths related to secondary malignant neoplasms of the bone in the United States from 1999 to 2023 [12]. Previous studies have used the CDC WONDER database to analyze trends in mortality due to primary malignant neoplasms of bone and cartilage [7]. Mortality due to secondary malignant bone neoplasms was identified by the International Classification of Diseases, 10th Revision (ICD-10) code C79.5 [13]. Individuals under 25 years of age were excluded, as secondary neoplasms of the bone are exceedingly rare in this age group. CDC WONDER provides aggregate mortality counts and corresponding population denominators rather than individual-level patient data. Data were reviewed for reportable values across each demographic and geographic group included in the analysis. Suppressed or unavailable subgroup data were not imputed, and analyses only included groups with reportable values. Per the CDC WONDER website, data are classed as “suppressed” for privacy reasons if a subgroup is small enough that there is a risk of an individual patient being identified. The threshold for suppression is set at 10 patients. Data is classed as unreliable if there are fewer than 20 deaths, as the relative standard error is greater than what is considered statistically reliable [12]. In our study, there were no data classed as suppressed, but data from several states (ex. Montana, Wyoming, District of Columbia) were classed as unreliable in some of the years. This study was exempt from institutional review board approval as the CDC WONDER database is composed of anonymized, publicly available data.

Demographic variables, including sex, race/ethnicity, age, urban/rural classification, census region, and state, were extracted. Chosen racial/ethnic group classifications, age groups, urban–rural areas, and census regions remained consistent with published CDC WONDER studies and the 2013 National Center for Health Statistics (NCHS) Urban-Rural Classification Scheme for Counties, which designates counties within the United States into one of six categories, ranging from large central metropolitan to noncore non-metropolitan [14].

Crude and age-adjusted mortality rates (AAMRs) per 100,000 US adults were calculated. Crude mortality rates were determined by dividing the number of secondary malignant neoplasms of bone-related deaths by the corresponding U.S. population. The U.S. standard population from 2000 was used in standardizing AAMRs as previously described [15]. Temporal trends were assessed using the Joinpoint Regression Program (version 5.4.0, available from the National Cancer Institute in Bethesda, Maryland), which models mortality trends throughout the study period [16]. The Joinpoint program creates linear models based on significant variation in annual mortality trends over time. Annual percentage changes (APCs) were then calculated for each generated linear segment. The Monte Carlo permutation test was used to compare models with different numbers of Joinpoints and determine whether adding a Joinpoint significantly improved model fit. Joinpoint models were selected using the program’s permutation-based model selection approach, which identifies significant changes in mortality trends. To further reduce overfitting, the maximum number of Joinpoints is determined by the Jointpoint Regression Program based on the number of annual data points input into the analysis software. The weighted average of APCs was calculated to give an average annual percentage change (AAPC), which summarizes mortality trends over the total study period. Statistical significance was set at *p* ≤ 0.05 (two-tailed test), with 95% confidence intervals reported.

## 3. Results

### 3.1. Overall Results

Between 1999 and 2023, 424,811 individuals in the United States over the age of 25 years had secondary malignant bone neoplasm-related mortality (Table S1).

**Figure 1:**
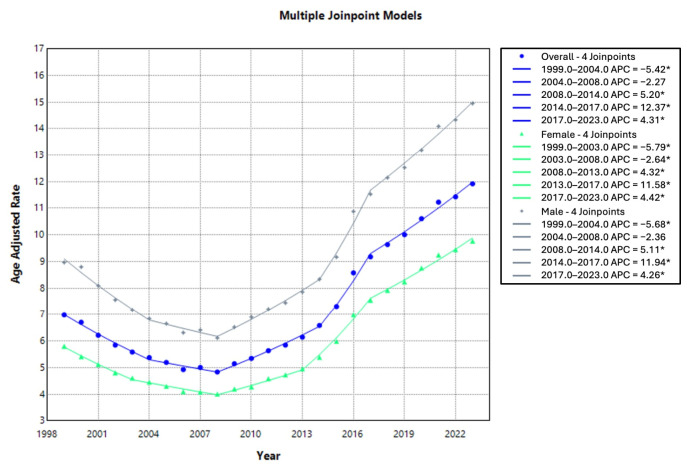
Joinpoint model of secondary bone neoplasm AAMR per 100,000 U.S. adults 25+ by sex, 1999–2023 (* APC significant).

The overall AAMR increased from 6.99 (95% CI 6.86 to 7.11) in 1999 to 11.92 (95% CI 11.79 to 12.05) in 2023. The AAPC over this period was 2.26 (95% CI 1.61 to 2.92). The lowest observed AAMR was 4.84 in 2008, and the highest observed AAMR was 11.92 in 2023. From 1999 to 2004, the APC in AAMR was −5.42 (95% CI −6.35 to −4.47), followed by a more gradual decrease in AAMR from 2004 to 2008. The AAMR overall increased from 2008 to 2023 with an APC of 12.37 (95% CI 8.18 to 16.72) between 2014 and 2017 (Figure 1).

### 3.2. Demographic Differences

#### 3.2.1. Gender Stratified

From 1999 to 2023, secondary malignant bone neoplasms were the cause of 189,999 (44.7%) deaths in females and 234,812 (55.3%) in males over the age of 25 years (Table S1). The AAMR increased in male individuals from 8.97 (95% CI 8.75 to 9.19) in 1999 to 14.95 (95% CI 14.73 to 15.17) in 2023, with an AAPC of 2.10 (95% CI 1.63 to 2.90). Between 1999 and 2004, the APC in AAMR was −5.68 (95% CI −6.85 to −4.50). From 2008 to 2014, the APC in AAMR became 5.11 (95% CI 3.85 to 6.38), accelerating further to 11.94 (95% CI 7.47 to 16.59) from 2014 to 2017. The APC slowed to 4.26 (95% CI 3.67 to 4.85) from 2017 to 2023 (Figure 1).

In female individuals, the AAMR overall increased from 5.81 (95% CI 5.66 to 5.96) in 1999 to 9.77 in 2023 (95% CI 9.61 to 9.94) with an AAPC of 2.26. Consistent with overall trends, AAMR decreased from 1999 to 2008, before increasing from 2008 to 2023. The APC was highest from 2013 to 2017 at 11.58 (95% CI 9.14 to 14.07).

#### 3.2.2. Race Stratified

**Figure 2:**
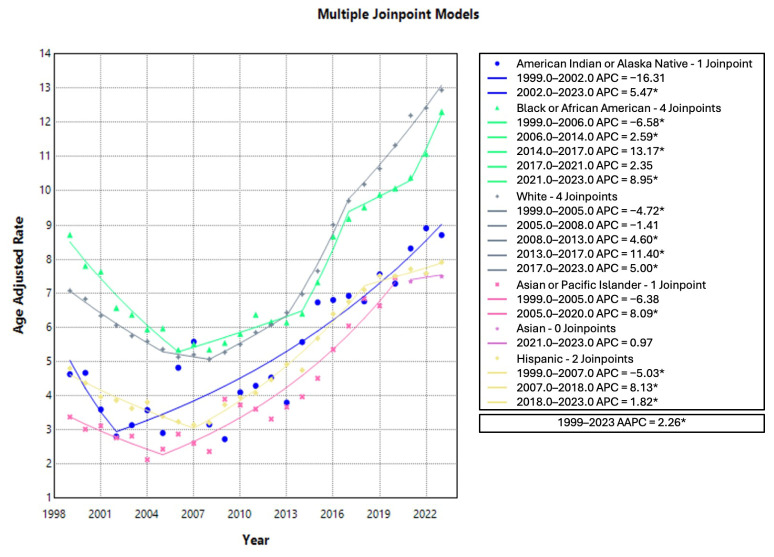
Joinpoint model of secondary bone neoplasm AAMR per 100,000 U.S. adults 25+ by race, 1999–2023 (* APC significant).

Black individuals had the highest AAMR from 1999 to 2012, with an AAMR of 8.72 (95% CI 8.25 to 9.19) in 1999 and 6.16 (95% CI 5.82 to 6.50) in 2012 (Table S2). This group had an overall AAPC of 1.53 (95% CI 0.11 to 2.97) from 1999 to 2023. Black individuals did have a decrease in AAMR between 1999 and 2006, followed by an increase over the rest of the study period, consistent with overall trends. From 2013 to 2023, White individuals had the highest AAMR, with an AAMR of 6.43 (95% CI 6.31 to 6.55) in 2013 and 12.94 (95% CI 12.78 to 13.11) in 2023, having an AAPC of 2.60 (95% CI 1.61 to 3.60) over the entire study period (Table S2). Following overall study trends, White individuals did have an initial decrease in AAMR from 1999 to 2008, followed by an increase from 2008 to 2023 (Figure 2).

Asian or Pacific Islanders were the group with the most significant increase in AAMR, increasing from 3.38 (95% CI 2.8 to 3.95) in 1999 to 7.45 (95% CI 7.0 to 7.91) in 2020, with an AAPC of 3.74 (95% CI 1.17 to 6.38) over this period (Table S2). Hispanic individuals also had a significant increase in AAMR from 4.8 (95% CI 4.34 to 5.27) in 1999 to 7.92 (95% CI 7.59 to 8.24) in 2023, with an AAPC of 2.26 (95% CI 1.51 to 3.02) (Table S2). The American Indian or Alaskan Native group had a non-significant increase in AAMR from 4.63 (95% CI 3.17 to 6.53) in 1999 to 8.71 (95% CI 7.32 to 10.09) in 2023, with an AAPC of 2.46 (95% CI −1.51 to 6.59) (Table S2).

#### 3.2.3. Age Groups Stratified

**Figure 3:**
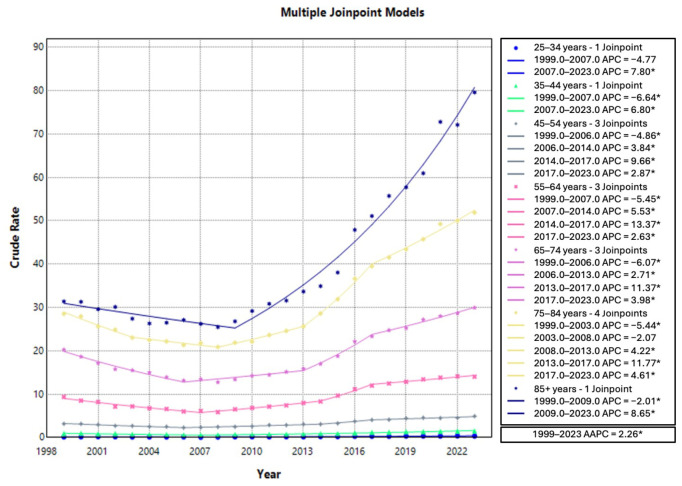
Joinpoint model of secondary bone neoplasm CMR per 100,000 U.S. adults 25+ by age groups, 1999–2023 (* APC significant).

All age groups had a significant increase in mortality rate over the study period. Crude mortality rates (CMRs) were used for age-stratified analyses to reflect age-specific mortality burden (Table S3). The population aged 85 years and older had the highest CMR, increasing from 31.9 (95% CI 29.69 to 33.1) in 1999 to 79.56 (95% CI 77.34 to 81.79) in 2023, with an AAPC of 4.77 (95% CI 3.38 to 4.77). From 1999 to 2009, the group did have a decrease in CMR followed by an increase from 2009 to 2023. Individuals aged 25 to 34 years had the next largest increase in CMR from 0.14 (95% CI 0.11 to 0.18) in 1999 to 0.34 (95% CI 0.29 to 0.40) in 2023, with an AAPC of 3.43 (95% CI 1.52 to 5.39). The 45- to 54-year cohort had the smallest increase in CMR from 3.17 (95% CI 2.99 to 3.35) in 1999 to 4.92 (95% CI 4.71 to 5.14) in 2023, with an AAPC of 1.68 (95% CI 0.64 to 2.73). All other age groups followed a similar trend of a decrease in crude mortality over approximately 10 years, followed by an increase until the end of the study (Figure 3).

### 3.3. Regional Variations

#### 3.3.1. Rural vs. Urban

**Figure 4:**
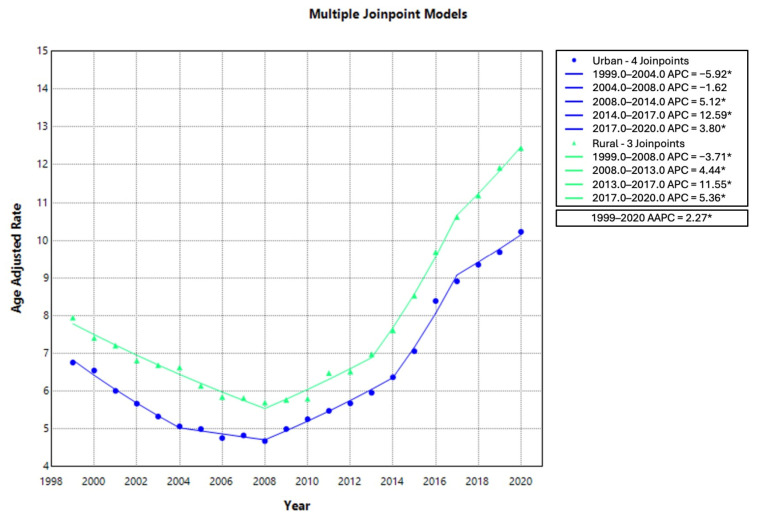
Joinpoint model of secondary bone neoplasm AAMR per 100,000 U.S. adults 25+ by urban vs. rural, 1999–2023 (* APC significant).

In comparing urban to rural regions, AAMRs were higher in rural areas than in urban areas from 1999 to 2020, though both saw overall increases in AAMR. In urban areas, AAMR increased from 6.76 (95% CI 6.63 to 6.90) in 1999 to 10.23 (95% CI 10.10 to 10.37) with an AAPC of 1.91 (95% CI 1.27 to 2.55). In rural areas, AAMR increased from 7.95 (95% CI 7.64 to 8.26) in 1999 to 12.44 (95% CI 12.10 to 12.78) in 2020 with an AAPC of 2.27 (95% CI 1.52 to 3.02). Both urban and rural regions initially showed a significant decrease in AAMR, followed by a significant increase (Figure 4, Table S4).

#### 3.3.2. State-Level Differences

**Figure 5:**
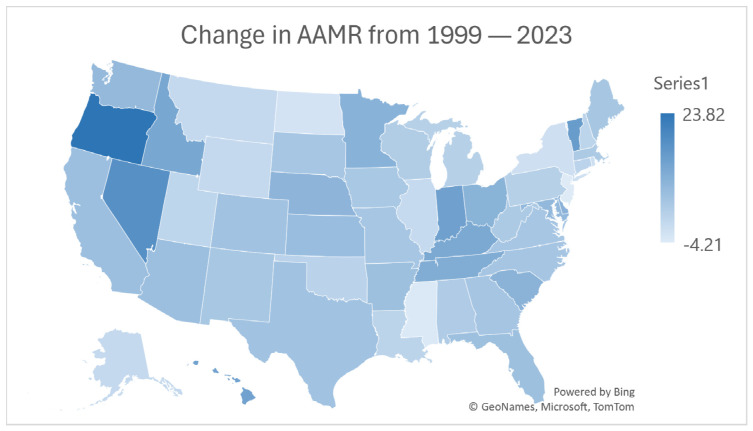
Joinpoint model of secondary bone neoplasm AAMR per 100,000 U.S. adults 25+ by state, 1999–2023.

The largest increase in AAMR occurred in Oregon from 4.58 in 1999 to 28.4 in 2023. While most states saw an increase in AAMR, several states, including Illinois, Mississippi, New Jersey, New York, North Dakota, and Rhode Island, had a decrease in AAMR. The largest decrease in AAMR occurred in New Jersey from 7.95 in 1999 to 3.74 in 2023 (Figure 5).

#### 3.3.3. Census Region-Based Differences

**Figure 6:**
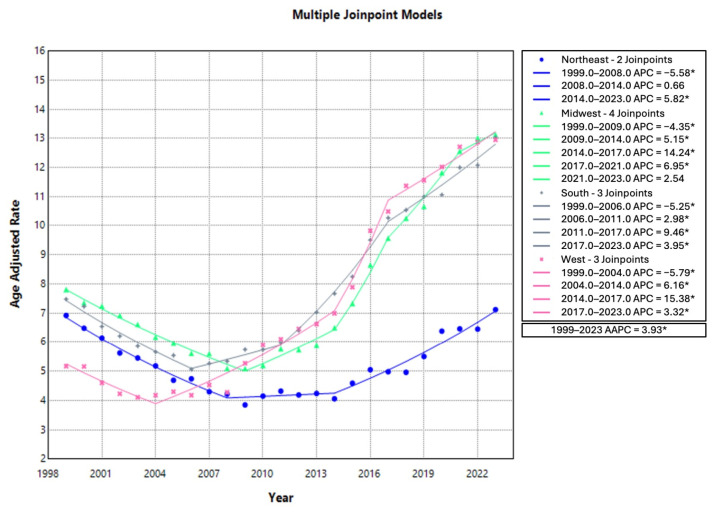
Joinpoint model of secondary bone neoplasm AAMR per 100,000 U.S. adults 25+ by census region, 1999–2023 (* APC significant).

The West, Midwest, and South census regions had a significant increase in AAMR over the study period after an initial decrease. The Northeast did not have a significant increase in AAMR. The Western region had the largest AAMR increase from 5.17 (95% CI 4.94 to 5.41) in 1999 to 12.95 (95% CI 12.67 to 13.24) in 2023, with an AAPC of 3.93 (95% CI 2.44 to 5.44). From 1999 to 2004, the AAMR in the West decreased to 4.18 (95% CI 3.98 to 4.38) with an APC of −5.79 (95% CI −9.13 to −2.33). From 2014 to 2017, AAMR in the West increased from 6.99 (95% CI 6.77 to 7.22) to 10.49 (95% CI 10.22 to 10.76) with an APC of 15.38 (95% CI 4.47 to 27.43). In the Midwest, AAMR increased from 7.81 (95% CI 7.54 to 8.08) in 1999 to 13.12 (95% 12.82 to 13.42) in 2023 with an AAPC of 2.21 (95% 1.20 to 3.22). In the South, AAMR increased from 7.48 (95% CI 7.26 to 7.69) in 1999 to 13.02 (95% CI 12.80 to 13.24) in 2023, with an AAPC of 2.29 (95% CI 1.59 to 2.99) (Figure 6, Table S5).

## 4. Discussion

In this population-based analysis of the CDC WONDER database, we found interesting trends in mortality, both overall and related to age, sex, race, and region. AAMR increased across groups, with adults over the age of 85 years, females, Asian and Pacific Islanders, and rural populations demonstrating the largest increases in AAMR.

Overall, AAMR increased from 1999 to 2023 at an AAPC of 2.26, though there was a period of decreasing AAMR from 1999 to 2008 where the APC was between −2.27 and −5.42. This pattern was observed across demographic subgroups. Cancer incidence and mortality have decreased over the past 20 years in the U.S., with improved survival being attributed to advancements in treatment [6,17]. However, the incidence of certain cancers, including breast and prostate cancer, increased in the U.S. from 2018 to 2022 [17]. Additionally, the incidence of cancers metastatic to the bone has increased globally [18,19]. Prior analyses of the CDC WONDER database have demonstrated increased AAMR from secondary malignancies in the United States between 1999 and 2019. Between 2013 and 2019, secondary bone malignancies accounted for the second largest increase in secondary neoplasms behind the adrenal glands [6]. Secondary malignancies, by definition, arise from the metastasis of a primary tumor. Breast, prostate, thyroid, lung, and bladder cancers are among the most common primary tumors that spread to the bone [20]. Overall, studies have found that lung cancer is the most common primary tumor metastasizing to bone, though rates of lung cancer have been decreasing since 1992 [17,21]. Although not definitively proven, we hypothesize that the increased incidence of primary cancers, including breast, prostate, and thyroid, might be linked to increased mortality from secondary malignancies of the bone. Incidence and survival rates of breast and prostate cancers have both improved, likely at least in part due to earlier detection and better treatment options. Though survival rates from these cancers increase due to better screening and treatment, incidence rates have also increased [17]. We suggest that the increase in mortality from secondary malignancies of the bone, despite increased survival rates of the primary malignancies, might be at least in part due to concomitant progression of aggressive primary malignancies. Fukutomi et al. suggested that as patients with hepatocellular carcinoma survived longer, there was a subsequent increase in metastasis to the bone [19]. Although further research is needed, we suggest that as therapies improve and patients survive for longer with their primary cancer, it is still possible that these cancers are still able to metastasize to the bone later in the disease course, especially if the therapies are not definitively curative. This could then contribute to higher rates of mortality attributed to secondary malignant neoplasms of the bone [19]. Additionally, studies have demonstrated a relationship between radiation and chemotherapy and the development of secondary neoplasms [6]. By the time cancers have metastasized to the bone, treatment options are typically more limited and focus on symptomatic relief rather than being curative, leading to lower overall survival rates [20]. Bisphosphonates, radiotherapy, and surgical interventions are some of the options [20,22]. However, survival rates for patients with bone metastases are low. Typically, median survival is 6–12 months, though it can be up to 25 months in breast cancer, 48 months in thyroid cancer, and 53 months in prostate cancer [20]. We hypothesize that more advanced primary cancers that have been treated by radiation and chemotherapy without a cure may eventually progress to secondary malignancies and ultimately lead to death.

The observed increase in secondary cancer mortality after 2009 could possibly reflect changes in diagnostic sensitivity over time. Diagnosis of secondary metastases of the bone has become increasingly easy due to advancements in detection and screening [22,23]. Secondary bone neoplasm diagnoses are occasionally due to incidental findings. CT scans have become increasingly common imaging tools in patients presenting with undifferentiated symptoms [22]. Patients occasionally are found to have lesions during work-up for unrelated issues. Additionally, patients may present with pathologic fractures without having a prior cancer diagnosis and be found to have metastatic bone lesions during work-up. Per Costa et al., up to 10% of patients without a prior cancer diagnosis might initially present with spinal metastases [24]. We suggest that some of the increase in deaths attributed to secondary malignant neoplasms of the bone might be related to increased detection of these neoplasms, which in turn allows for secondary malignant neoplasms of the bone to be listed as a diagnosis on death certificates. Newer technologies, such as whole-body MRI, SPECT/CT, and PET/CT, allow for both detection and staging of malignancies that might have been more difficult to detect in the past [23]. We hypothesize that due to increased availability of more sensitive screening modalities, more secondary neoplasms of the bone could be detected. Centers for Medicare and Medicaid Services revised its national FDG-PET coverage framework effective for claims on or after 3 April 2009, replacing the prior diagnosis/staging/restaging/monitoring framework with a broader initial and subsequent antitumor treatment strategy framework; CMS also determined that FDG-PET was useful for determining the appropriate initial treatment strategy for beneficiaries with suspected solid tumors and myeloma [25]. Hillner et al. similarly reported that CMS expanded PET coverage in April 2009 for the initial treatment strategy of most solid tumors, supporting the possibility that increased PET use contributed to greater detection and documentation of metastatic disease [25]. Although bone metastases can be identified easily, there are few curative treatment options currently available, raising the incidence rate while mortality stays the same. Observed increases may reflect improvements in detection rather than true changes in underlying disease burden. However, we hypothesize that increased detection of bone metastasis may inflate observed AAMR estimates without reflecting a true or proportional increase in underlying disease burden. Observed increases in AAMR may reflect improvements in detection of bone metastasis without reflecting a true or proportional increase in underlying disease burden.

Additionally, cancer screenings became increasingly common and accurate over the study period, which we suggest could have potentially led to an increase in detection of primary cancers and a subsequent increase in discovery of secondary malignancies of the bone. Although screening guidelines for common cancers, including breast cancer, have been used since the 1960s and 1970s, the U.S. Preventive Services Task Force has been instrumental in implementing clearer, more rigorous screening guidelines since 1984 [26,27]. These guidelines are updated annually based on new screening modalities [26]. It is possible that increased screening for primary malignancies during this period may contribute to the observed rise in secondary bone malignancies. We propose two possible reasons for this. Firstly, primary cancers might have been identified on screening, prompting further work-up and identification of secondary cancers. Secondly, primary cancers might have been diagnosed earlier in the study period due to increased screening and treated at the time, with metastases developing later in the study period. Further studies would be needed to definitively link increased cancer screenings to increased discovery of secondary malignancies.

Interestingly, the AAMR from secondary bone neoplasms was lower in the 1999–2009 period. Overall, cancer incidence and death rates did decrease over this period [17]. Lower observed rates may reflect earlier mortality from primary cancers prior to progression. We propose that individuals might have died earlier in the disease course, prior to developing metastases. As an alternative hypothesis, it is possible that bone lesions were being erroneously identified as primary cancers rather than metastases from previously known cancers due to limitations in screening and less accurate imaging modalities [20,22]. This discrepancy could be related to differences in primary cancer type. Vandecandelaere et al., in a study of two cohorts of patients with secondary bone neoplasms, found that the 1989–1996 cohort had a greater increase in the percentage of lung cancer patients presenting with metastatic disease compared to the 1958–1967 cohort. In contrast, the percentage of breast and prostate cancer with bony metastases was decreased in the 1989–1996 cohort compared to the 1958–1967 cohort [28]. We hypothesize that more individuals in the 1999–2009 period of our study had primary cancers that were less likely to metastasize to the bone, while individuals in the 2009–2023 period had primary cancers that commonly metastasize to the bone.

Both sexes saw increases in AAMR over the study periods, with an AAPC of 2.10 in male individuals and 2.26 in female individuals. Though men in our study had higher AAMRs overall, the AAPC was higher for females. Spinal metastases overall appear to occur at higher rates in males [18,21,24]. Males have higher overall rates of cancer compared to females, with prostate cancer being more common than breast cancer [17,24]. These patterns may reflect differences in primary cancer type or treatment response between sexes. Median survival in metastatic prostate cancer patients tends to be 12–53 months, while in metastatic breast cancer patients, it is 19–25 months [20]. Additionally, it is possible that rates of metastatic breast cancer are now increasing at higher rates than metastatic prostate cancer, leading to a greater increase in AAPC among females. However, as we do not have access to data on primary cancers in these patients, we cannot speculate further as to whether this impacted sexual differences in AAMR.

Mortality rates were highest in Black individuals in our study from 1999 to 2012, consistent with prior studies of secondary malignancies [6,29]. However, overall rates of cancer were highest among non-Hispanic White individuals from 2000 to 2022, followed by non-Hispanic American Indian/Alaska Natives, non-Hispanic Black, and Hispanic individuals [17]. Prior studies have shown that Black and other minority patients receive worse oncologic care and have poorer outcomes. These differences, however, cannot be fully attributed to race alone [29]. These findings highlight persistent racial disparities in outcomes, warranting further investigation into structural and healthcare system-level factors. These disparities may reflect differences in cancer screening and treatment access due to socioeconomic factors, comorbidities, and broader structural factors. Interestingly, other studies of bone metastasis have shown worse overall survival in White patients [30]. In our study, White individuals did have the highest AAMR between 2013 and 2023. Notably, Asian or Pacific Islander individuals had the greatest increase in AAPC over the study period. Xu et al. report that Asian Americans and Pacific Islanders developed bone metastases at higher rates than White and Black patients, though they had the longest overall survival [31]. The reason for this variation by race remains unclear, but based on prior studies, we suggest that other characteristics, including socioeconomic status and health inequity, might play a role in the development of metastatic bone malignancy. Future studies should focus on addressing these disparities.

Though all age groups in our study saw an increase in mortality rate over the entire study period, the largest increase in mortality rate occurred in individuals over 85 years. This is overall unsurprising as cancer deaths generally increase with age. Prior studies have shown that patients typically develop secondary malignancies of the bone between 50 and 70 years of age [18,21,24,28]. These patients likely developed and were treated for primary cancers earlier in life. Treatment of cancers by radiation has been associated with the development of secondary malignancies [6]. An alternative hypothesis for the increased mortality in this age group is that these patients might have developed primary cancers later in life and been ineligible for treatment due to functional status or elected not to pursue treatment given a limited life expectancy. They also might have developed primary cancer after stopping cancer screenings. Cancer screenings generally stop at age 75 unless patients have a life expectancy of another 10 years [32]. Older patients are also more prone to falls, which can lead to fractures, especially in bones with metastatic lesions, which we suggest might have further contributed to increased mortality rates. These patterns likely reflect a convergence of healthcare accessibility and comorbid disease burden, rather than a single causal mechanism. Overall, there are many likely contributors to increased rates of mortality among older patients with secondary malignancies of the bone.

Throughout the study period, AAMRs were higher in rural areas compared to urban areas. This is consistent with the 2013 NCHS classification data, which showed that mortality across age groups was generally higher in more rural areas compared to urban areas [14]. Similarly, the overall health score as well as the percent insured was lower in rural areas compared to urban areas. Although the CDC WONDER database does not provide data on social determinants of health and comorbidities, the 2013 NCHS data does suggest that rural areas might be affected by some of these confounding factors. We hypothesize that patients in rural areas might have had less access to insurance and have been in worse health at baseline compared to their urban counterparts. We propose that patients in rural areas diagnosed with secondary malignant neoplasms of the bone may have struggled to obtain access to specialized care due to distance from health centers or other social determinants of health, including lower income or lack of insurance. All of these barriers could lead to diagnosis later in the disease course or delays in obtaining treatment. If rural patients did seek treatment, they might have received suboptimal treatment compared to urban patients. In one study, Mak et al. found that patients who presented to urban hospitals with malignant spinal cord compression were more likely to receive surgery or radiotherapy compared to patients who presented to rural hospitals. They also found increased survival rates among patients who received surgery [33]. We suggest that patients in rural areas might not have access to larger health centers with interdisciplinary teams, which could potentially limit treatment options and timing of treatment. All of these confounding factors could have potentially contributed to higher AAMRs among rural populations.

Our study did have several limitations. The chief limitation is that, due to this being an observational database study, the results are based upon previously input data that cannot be independently verified or fully analyzed for confounding factors. We also lack data on factors that might have impacted mortality, such as the site of the primary tumor, comorbid conditions, and treatment course. Because we do not know the primary cancer type, we are unable to definitively attribute the rise in bone metastases to a specific type of cancer. The CDC Wonder data does not provide primary tumor site, stage at diagnosis, modality for imaging used, timing of metastatic spread, interval from bone metastasis to death, systemic therapy, or other clinical/treatment data. This is a fundamental limitation of this study as it limits our analysis and hypothesis of notable trends, such as the sharp increase in AAMR after 2009. Access to further clinical and treatment data for patients may help identify if there is a true rise in mortality from secondary malignant neoplasm of the bone or if there is another explanation for the trends detected in this study. We furthermore do not know whether the primary or metastatic cancer was diagnosed first, which has implications for prognosis, as patients with metastatic disease are more advanced and typically do not respond as well to treatment. Additionally, the exact incidence of bone metastasis is difficult to quantify [20]. Patients may present with bony lesions late in the disease course after they have elected to stop treatment and are no longer undergoing imaging. We also cannot determine the exact cause of mortality in patients. Furthermore, the CDC WONDER database does not provide data on other comorbidities or socioeconomic status. Both underlying health issues and socioeconomic status can affect access to and efficacy of healthcare and are, therefore, important confounding factors when considering mortality that are unfortunately not included in our analysis, though it is possible that they would have affected AAMR. Additionally, in the analysis of individual states, data from several states (ex., Montana, Wyoming, District of Columbia) were classed as unreliable in some of the years. Data is classed as unreliable if there are fewer than 20 deaths because the relative standard error is considered statistically unreliable [12]. Tiwari et al. found that excluding this data often leads to an underestimate of mortality in small populations [34]. It is possible that several states, such as Montana and Wyoming, might have had higher AAMRs relative to their population size, but this unfortunately could not be calculated due to unreliable data. Another limitation of the CDC WONDER database is its reliance on death certificates. Prior studies have found that the manner of death may be inaccurately reported on death certificates or that other major contributing factors may not be reported [35]. Although the CDC WONDER database reports underlying cause of death and multiple contributing causes of death, we cannot be sure whether secondary malignant neoplasms of the bone were accurately reported as contributors on death certificates. Variations in death certification and coding likely impact trends in mortality as the CDC WONDER database depends on reported data rather than clinical findings [36]. It is possible that in some instances secondary malignant neoplasms of the bone were not reported despite being significant contributors, or that in other instances they were reported despite not being significant. The lack of consistency in coding and reporting on death certificates could affect reported numbers of deaths attributed to secondary malignant neoplasms of the bone. Additionally, the risk of misclassification due to ICD-10 codes is a weakness in CDC WONDER studies. We queried for a single ICD-10 code (C79.5-secondary malignant neoplasm of bone and bone marrow). However, we cannot be certain that all of the malignancies related to the death had actually metastasized to become secondary malignant neoplasms of the bone and bone marrow. For instance, they might have been primary malignant neoplasms of bone and articular cartilage (ICD-10 code C40-41), which were erroneously classified as secondary. We also did not account for neoplasms that had unknown primary sites (ICD-10 code C80 malignant neoplasm without specification of site), although it is possible that these neoplasms had metastasized to the bone. In order to ensure that our analysis did not include patients without secondary malignant neoplasms of the bone and bone marrow, we chose to use a singular ICD-10 code, but we recognize that in doing so, there is a risk of misclassification bias. Prior studies have shown that misclassification by ICD code and death certificate can have significant impacts on national databases like CDC WONDER [35,37]. McGivern et al. found that errors in cause of death classification would have impacted national databases, though reasons, such as different interpretations by individual providers, behind the misclassification varied. In some cases, the coding after death certification contributed to error [37]. It is likely that this CDC WONDER analysis was affected by similar misclassification bias, especially because it encompasses many healthcare systems over a large time period. This misclassification bias could have impacted how many deaths were correctly attributed to secondary malignant neoplasms of the bone. Furthermore, although death is attributed to the bone metastases, we do not have access to the specific cause, such as pathological fracture, bone marrow suppression, or hypercalcemia [23]. Finally, we do not know the timeline from detection of the metastases to death or what interventions patients underwent. Both of these factors could have impacted disease progression and survival rates.

Despite these limitations, our study had many strengths. While prior studies have focused on metastases from specific primary cancers, we instead focused on overall metastases to the bone [22]. Many prior studies emphasize primary tumor of origin, age at diagnosis, and survival rates, but we have examined overall trends in mortality regardless of primary tumor. Additionally, this study is unique in that it demonstrates considerable variation in AAMR by demographic group.

## 5. Conclusions

This study is important because it demonstrates potential future directions for the treatment of secondary malignant neoplasms of the bone. The trends in our study should help inform screening guidelines for patients who are at risk of demonstrating bony metastases. Earlier detection may inform care planning and risk analysis, though impacts on survival cannot be determined from mortality data alone [6,23]. As a descriptive, population-level mortality analysis, this study identifies trends and disparities but cannot establish causal relationships. Future studies should focus on the prevention of primary malignancies and minimizing risk factors for the development of secondary malignancies. Hopefully, with new advancements in technology, more curative options will be developed.

## Data Availability

The data was accessed and collected on 12 February 2026, from the CDC WONDER database, which includes clinical and genomic data from 1999 to 2023.

## Supplementary Materials

The following supporting information can be downloaded at: https://www.mdpi.com/article/10.3390/cancers18121877/s1, Table S1: Annual deaths and age-adjusted mortality rate (AAMR) overall and by sex from 1999 to 2023, Table S2: Age-adjusted mortality rate (AAMR) by race and ethnicity from 1999 to 2023, Table S3: Crude mortality rate by age group from 1999 to 2023, Table S4: Age-adjusted mortality rate (AAMR) compared by urban versus rural regions from 1999 to 2023, Table S5: Age-adjusted mortality rate (AAMR) by census region from 1999 to 2023.

## Abbreviations

The following abbreviations are used in this manuscript:

CDC WONDER	Centers for Disease Control and Prevention Wide-ranging ONline Data for Epidemiologic Research
PET-CT	Positron emission tomography–computed tomography
SPECT/CT	Single-photon emission computed tomography/computed tomography
MRI	Magnetic resonance imaging
SRE	Skeletal-related event
ICD-10	International Classification of Diseases, 10th Revision
NCHS	National Center for Health Statistics
AAMR	Age-adjusted mortality rate
APC	Annual percentage change
AAPC	Average annual percentage change
CI	Confidence interval
