# Supplementary material for: Trends and Disparities in Secondary Malignant Neoplasms of the Bone in the United States: The WONDER Study
Source: Cancers (Basel). 2026 Jun 9;18(12):1877. doi: 10.3390/cancers18121877 (PMC13297235; doi:10.3390/cancers18121877)
Supplement: Supplementary file 1 [file cancers-18-01877-s001.zip › cancers-4294440-supplementary.pdf]

Supplemental Table S1. Annual Deaths and Age Adjusted Mortality Rate (AAMR) Overall and by Sex from 1999-2023

Year	Overall Annual Deaths	Overall AAMR	Overall 95% Confidence Interval	Female Annual Deaths	Female AAMR	Female 95% Confidence Intervals	Male Annual Deaths	Male AAMR	Male 95% Confidence Intervals
1999	12377	6.99	[6.86, 7.11]	5778	5.81	[5.66, 5.96]	6599	8.97	[8.75, 9.19]
2000	11981	6.71	[6.59, 6.83]	5459	5.42	[5.27, 5.56]	6522	8.8	[8.58, 9.02]
2001	11346	6.22	[6.1, 6.33]	5219	5.12	[4.98, 5.26]	6127	8.1	[7.89, 8.3]
2002	10850	5.85	[5.74, 5.96]	5021	4.81	[4.68, 4.94]	5829	7.56	[7.37, 7.76]
2003	10560	5.59	[5.48, 5.7]	4869	4.61	[4.48, 4.74]	5691	7.18	[6.99, 7.37]
2004	10305	5.38	[5.28, 5.48]	4773	4.46	[4.33, 4.59]	5532	6.85	[6.66, 7.03]
2005	10209	5.2	[5.1, 5.3]	4733	4.3	[4.17, 4.42]	5476	6.66	[6.48, 6.84]
2006	9860	4.93	[4.83, 5.03]	4550	4.1	[3.98, 4.22]	5310	6.32	[6.15, 6.5]
2007	10162	5.01	[4.92, 5.11]	4616	4.08	[3.96, 4.2]	5546	6.42	[6.25, 6.6]
2008	10057	4.84	[4.74, 4.93]	4616	4.01	[3.89, 4.12]	5441	6.12	[5.96, 6.29]
2009	10901	5.15	[5.05, 5.24]	4941	4.2	[4.08, 4.32]	5960	6.53	[6.37, 6.7]
2010	11581	5.35	[5.25, 5.44]	5130	4.28	[4.17, 4.4]	6451	6.91	[6.73, 7.08]
2011	12515	5.64	[5.54, 5.74]	5615	4.59	[4.47, 4.71]	6900	7.21	[7.04, 7.38]
2012	13278	5.85	[5.75, 5.95]	5907	4.73	[4.61, 4.86]	7371	7.45	[7.28, 7.62]
2013	14341	6.15	[6.05, 6.25]	6315	4.96	[4.84, 5.09]	8026	7.86	[7.68, 8.03]
2014	15678	6.59	[6.49, 6.7]	6979	5.39	[5.27, 5.52]	8699	8.34	[8.16, 8.52]
2015	17828	7.31	[7.2, 7.42]	7939	5.99	[5.86, 6.1]	9889	9.17	[8.99, 9.36]
2016	21415	8.58	[8.47, 8.7]	9453	6.99	[6.85, 7.13]	11962	10.88	[10.68, 11.08]
2017	23432	9.18	[9.06, 9.3]	10415	7.55	[7.4, 7.7]	13017	11.53	[11.33, 11.74]
2018	25209	9.64	[9.52, 9.76]	11138	7.92	[7.77, 8.07]	14071	12.15	[11.95, 12.36]

2019	26672	10.01	[9.89, 10.14]	11796	8.24	[8.09, 8.4]	14876	12.53	[12.33, 12.74]
2020	28701	10.61	[10.49, 10.74]	12717	8.76	[8.61, 8.92]	15984	13.19	[12.98, 13.39]
2021	30286	11.23	[11.1, 11.36]	13343	9.25	[9.09, 9.41]	16943	14.09	[13.88, 14.31]
2022	31724	11.43	[11.3, 11.56]	14024	9.45	[9.29, 9.61]	17700	14.33	[14.12, 14.55]
2023	33543	11.92	[11.79, 12.05]	14653	9.77	[9.61, 9.94]	18890	14.95	[14.73, 15.17]

Supplemental Table S2. Age Adjusted Mortality Rate (AAMR) by Race and Ethnicity from 1999-2023

Year	American Indian or Alaska Native AAMR	American Indian or Alaska Native 95% Confidence Interval		Black or African American AAMR	Black or African American 95% Confidence Interval		White AAMR	White 95% Confidence Interval		Asian or Pacific Islander AAMR	Asian or Pacific Islander 95% Confidence Interval		Hispanic AAMR	Hispanic 95% Confidence Interval	
1999	4.63	3.17	6.53	8.72	8.25	9.19	7.07	6.94	7.21	3.38	2.8	3.95	4.8	4.34	5.27
2000	4.67	3.28	6.43	7.81	7.37	8.25	6.83	6.7	6.97	3.02	2.53	3.52	4.37	3.94	4.81
2001	3.6	2.41	5.16	7.64	7.21	8.07	6.34	6.21	6.47	3.12	2.61	3.63	3.97	3.57	4.36
2002	2.81	1.78	4.21	6.57	6.17	6.97	6.06	5.94	6.19	2.77	2.32	3.23	3.87	3.48	4.25
2003	3.14	2.07	4.56	6.37	5.99	6.76	5.75	5.63	5.87	2.82	2.37	3.27	3.63	3.27	3.98
2004	3.58	2.48	5.01	5.94	5.57	6.31	5.59	5.47	5.71	2.13	1.75	2.5	3.81	3.44	4.17
2005	2.91	1.95	4.18	5.97	5.6	6.33	5.36	5.25	5.48	2.44	2.05	2.83	3.4	3.07	3.72
2006	4.82	3.49	6.49	5.34	5	5.69	5.13	5.02	5.24	2.88	2.46	3.3	3.24	2.92	3.56
2007	5.58	4.12	7.37	5.51	5.17	5.86	5.2	5.09	5.32	2.61	2.22	2.99	3.15	2.85	3.45
2008	3.16	2.19	4.41	5.35	5.01	5.68	5.07	4.96	5.18	2.37	2.02	2.72	3.21	2.91	3.5
2009	2.73	1.83	3.91	5.55	5.21	5.88	5.27	5.16	5.38	3.9	3.46	4.34	3.74	3.43	4.05
2010	4.1	3.01	5.45	5.81	5.47	6.15	5.5	5.39	5.62	3.73	3.3	4.15	3.95	3.64	4.26

2011	4.29	3.17	5.67	6.37	6.02	6.72	5.85	5.73	5.97	3.61	3.21	4	4.08	3.76	4.39
2012	4.53	3.38	5.94	6.16	5.82	6.5	6.07	5.96	6.19	3.32	2.95	3.69	4.47	4.16	4.78
2013	3.8	2.83	5	6.14	5.81	6.47	6.43	6.31	6.55	3.67	3.29	4.06	4.92	4.6	5.24
2014	5.57	4.33	7.05	6.4	6.07	6.74	6.97	6.84	7.09	3.97	3.59	4.36	4.75	4.45	5.05
2015	6.73	5.4	8.29	7.32	6.97	7.67	7.66	7.53	7.79	4.51	4.11	4.9	5.68	5.35	6
2016	6.8	5.43	8.17	8.67	8.29	9.05	9.01	8.87	9.15	5.35	4.93	5.77	6.39	6.05	6.72
2017	6.92	5.58	8.27	9.19	8.81	9.57	9.71	9.56	9.85	6.04	5.61	6.48	6.75	6.41	7.09
2018	6.76	5.5	8.03	9.52	9.13	9.9	10.19	10.05	10.34	6.84	6.38	7.29	7.1	6.76	7.44
2019	7.56	6.24	8.89	9.89	9.5	10.28	10.65	10.5	10.8	6.63	6.19	7.07	7.52	7.17	7.86
2020	7.28	5.99	8.56	10.07	9.69	10.45	11.33	11.18	11.48	7.45	7	7.91	7.51	7.18	7.85
2021	8.32	6.89	9.75	10.39	10	10.78	12.2	12.04	12.36	7.34	6.88	7.8	7.72	7.38	8.06
2022	8.91	7.45	10.36	11.08	10.68	11.48	12.42	12.26	12.57	7.57	7.12	8.01	7.59	7.27	7.92
2023	8.71	7.32	10.09	12.31	11.89	12.74	12.94	12.78	13.11	7.49	7.05	7.93	7.92	7.59	8.24

Supplemental Table S3. Crude Mortality Rate by Age Group from 1999-2023

Year	25-34 Year Old Crude Mortality Rate	25-35 Year Old 95% Confidence Interval		35-44 Year Old Crude Mortality Rate	35-44 Year Old 95% Confidence Interval		45-54 Year Old Crude Mortality Rate	45-54 Year Old 95% Confidence Interval		55-64 Year Old Crude Mortality Rate	55-64 Year Old 95% Confidence Interval		65-74 Year Old Crude Mortalit	65-74 Year Old 95% Confidence Interval		75-84 Year Old Crude Mortality Rate	75-84 Year Old 95% Confidence Interval		85+ Year Old Crude Mortality Rate	85+ Year Old 95% Confidence Interval	
1999	0.14	0.11	0.18	0.91	0.82	0.99	3.17	2.99	3.35	9.37	8.99	9.76	20.26	19.61	20.91	28.53	27.59	29.48	31.39	29.69	33.1

2000	0.17	0.13	0.22	0.98	0.89	1.07	3.16	2.98	3.34	8.52	8.16	8.89	18.66	18.03	19.28	27.95	27.02	28.88	31.3	29.62	32.98
2001	0.2	0.15	0.24	0.84	0.75	0.92	2.99	2.82	3.16	8.21	7.85	8.56	17.16	16.56	17.76	25.61	24.72	26.49	29.57	27.94	31.19
2002	0.18	0.14	0.22	0.91	0.82	1	2.71	2.55	2.87	7.13	6.81	7.45	15.77	15.19	16.34	24.86	23.99	25.72	30.12	28.5	31.75
2003	0.13	0.1	0.17	0.75	0.67	0.84	2.69	2.53	2.85	7.19	6.88	7.5	15.51	14.95	16.08	23.03	22.2	23.86	27.41	25.87	28.94
2004	0.14	0.1	0.18	0.66	0.58	0.73	2.55	2.4	2.7	6.76	6.46	7.06	15.01	14.45	15.57	22.49	21.68	23.31	26.31	24.82	27.8
2005	0.13	0.1	0.17	0.7	0.62	0.78	2.5	2.35	2.65	6.59	6.3	6.88	13.92	13.39	14.45	22.18	21.37	22.99	26.48	25.01	27.96
2006	0.14	0.11	0.19	0.59	0.51	0.66	2.28	2.14	2.43	6.02	5.75	6.29	13.14	12.63	13.66	21.35	20.56	22.14	27.11	25.64	28.57
2007	0.08	0.06	0.12	0.56	0.49	0.63	2.36	2.21	2.5	6.13	5.86	6.39	13.46	12.95	13.98	21.79	20.99	22.59	26.21	24.8	27.63
2008	0.11	0.08	0.15	0.58	0.5	0.65	2.45	2.3	2.59	5.87	5.62	6.13	12.78	12.29	13.27	20.87	20.09	21.65	25.48	24.11	26.85
2009	0.16	0.12	0.2	0.68	0.6	0.76	2.48	2.33	2.62	6.52	6.25	6.78	13.41	12.92	13.9	21.88	21.08	22.69	26.79	25.41	28.18
2010	0.18	0.14	0.23	0.64	0.56	0.71	2.58	2.43	2.72	6.84	6.57	7.11	14.27	13.77	14.77	22.11	21.3	22.92	29.18	27.75	30.61
2011	0.17	0.13	0.22	0.74	0.66	0.83	2.88	2.72	3.04	7.14	6.87	7.41	14.47	13.98	14.97	23.63	22.8	24.46	30.83	29.4	32.27
2012	0.18	0.15	0.23	0.76	0.68	0.85	2.87	2.71	3.03	7.42	7.15	7.69	15.15	14.65	15.64	24.6	23.76	25.44	31.59	30.16	33.03
2013	0.16	0.12	0.2	0.74	0.66	0.83	3.08	2.92	3.25	7.99	7.71	8.27	15.88	15.39	16.37	25.62	24.76	26.48	33.67	32.21	35.13
2014	0.23	0.18	0.27	0.89	0.8	0.98	3.08	2.92	3.25	8.31	8.02	8.59	16.99	16.49	17.49	28.62	27.72	29.52	34.91	33.43	36.38
2015	0.22	0.17	0.26	0.9	0.81	1	3.26	3.09	3.43	9.67	9.36	9.97	18.78	18.26	19.29	31.9	30.96	32.83	38.05	36.52	39.57
2016	0.3	0.25	0.35	1.07	0.97	1.18	3.76	3.58	3.94	11.14	10.82	11.47	22.17	21.63	22.72	36.63	35.64	37.63	47.88	46.18	49.58
2017	0.3	0.25	0.35	1.22	1.11	1.32	4.07	3.88	4.26	11.98	11.65	12.31	23.34	22.79	23.89	39.5	38.48	40.52	51.08	49.34	52.82
2018	0.3	0.25	0.35	1.2	1.1	1.31	4.13	3.93	4.32	12.47	12.13	12.8	24.75	24.19	25.31	41.54	40.52	42.56	55.7	53.89	57.5
2019	0.34	0.29	0.39	1.34	1.23	1.46	4.43	4.22	4.63	12.86	12.52	13.2	25.23	24.67	25.78	43.41	42.38	44.43	57.71	55.88	59.55
2020	0.35	0.3	0.41	1.36	1.24	1.47	4.57	4.36	4.78	13.42	13.07	13.77	27.2	26.63	27.77	45.72	44.69	46.76	60.93	59.06	62.81

2021	0.33	0.28	0.39	1.44	1.33	1.56	4.49	4.28	4.7	13.83	13.48	14.19	28.01	27.45	28.58	49.23	48.15	50.31	72.79	70.63	74.96
2022	0.37	0.32	0.43	1.49	1.38	1.6	4.55	4.34	4.75	14.12	13.76	14.48	28.66	28.09	29.23	50	48.96	51.05	72.11	70.04	74.18
2023	0.34	0.29	0.4	1.5	1.39	1.62	4.92	4.71	5.14	14.04	13.68	14.4	29.95	29.38	30.53	51.88	50.84	52.92	79.56	77.34	81.79

Supplemental Table S4: Age Adjusted Mortality Rate (AAMR) Compared by Urban versus Rural Regions from 1999-2023

Year	Urban AAMR	Urban 95% Confidence Interval		Rural AAMR	Rural 95% Confidence Interval	
1999	6.76	6.63	6.9	7.95	7.64	8.26
2000	6.55	6.42	6.69	7.41	7.11	7.7
2001	6.01	5.88	6.13	7.21	6.92	7.5
2002	5.67	5.55	5.79	6.81	6.53	7.09
2003	5.33	5.22	5.45	6.69	6.41	6.96
2004	5.07	4.96	5.18	6.63	6.36	6.9
2005	5	4.89	5.11	6.14	5.88	6.4
2006	4.76	4.66	4.87	5.85	5.6	6.1
2007	4.83	4.72	4.93	5.82	5.57	6.07
2008	4.68	4.58	4.78	5.7	5.45	5.95
2009	5	4.89	5.1	5.77	5.52	6.01
2010	5.26	5.15	5.37	5.8	5.55	6.04
2011	5.48	5.38	5.59	6.48	6.22	6.74
2012	5.68	5.57	5.79	6.52	6.26	6.78
2013	5.96	5.85	6.07	6.98	6.72	7.25
2014	6.37	6.26	6.49	7.61	7.34	7.89

2015	7.06	6.95	7.18	8.53	8.24	8.82
2016	8.39	8.27	8.52	9.69	9.38	10
2017	8.91	8.78	9.04	10.62	10.3	10.94
2018	9.35	9.22	9.48	11.19	10.87	11.52
2019	9.69	9.56	9.82	11.92	11.58	12.25
2020	10.23	10.1	10.37	12.44	12.1	12.78

Supplemental Table S5: Age Adjusted Mortality Rate (AAMR) by Census Region from 1999-2023

Year	Northeast AAMR	Northeast 95% Confidence Interval		Midwest AAMR	Midwest 95% Confidence Interval		South AAMR	South 95% Confidence Interval		West AAMR	West 95% Confidence Interval	
1999	6.92	6.65	7.19	7.81	7.54	8.08	7.48	7.26	7.69	5.17	4.94	5.41
2000	6.48	6.22	6.73	7.35	7.09	7.61	7.23	7.02	7.44	5.16	4.92	5.39
2001	6.14	5.88	6.39	7.24	6.99	7.5	6.54	6.34	6.74	4.6	4.38	4.81
2002	5.62	5.38	5.86	6.92	6.67	7.17	6.21	6.02	6.4	4.23	4.03	4.44
2003	5.45	5.21	5.68	6.61	6.37	6.85	5.86	5.68	6.04	4.11	3.91	4.31
2004	5.18	4.96	5.41	6.17	5.94	6.4	5.66	5.48	5.84	4.18	3.98	4.38
2005	4.69	4.48	4.91	5.96	5.73	6.19	5.54	5.36	5.71	4.3	4.1	4.5
2006	4.74	4.52	4.95	5.61	5.39	5.82	5.06	4.9	5.23	4.18	3.98	4.38
2007	4.3	4.1	4.51	5.6	5.39	5.82	5.26	5.1	5.43	4.53	4.32	4.73
2008	4.22	4.02	4.42	5.09	4.88	5.29	5.34	5.17	5.5	4.28	4.09	4.48
2009	3.85	3.66	4.04	5.09	4.89	5.29	5.74	5.57	5.91	5.27	5.06	5.49
2010	4.15	3.96	4.35	5.2	4.99	5.4	5.74	5.57	5.9	5.9	5.68	6.12
2011	4.32	4.12	4.52	5.77	5.56	5.98	5.97	5.8	6.14	6.1	5.88	6.33

2012	4.19	3.99	4.38	5.73	5.52	5.95	6.4	6.23	6.57	6.46	6.23	6.68
2013	4.24	4.04	4.43	5.89	5.68	6.1	7.03	6.85	7.2	6.62	6.39	6.84
2014	4.06	3.87	4.25	6.5	6.27	6.72	7.67	7.49	7.85	6.99	6.77	7.22
2015	4.59	4.39	4.79	7.33	7.09	7.56	8.25	8.06	8.44	7.89	7.65	8.13
2016	5.05	4.84	5.26	8.65	8.4	8.9	9.51	9.31	9.71	9.83	9.57	10.1
2017	4.98	4.77	5.18	9.57	9.3	9.83	10.27	10.07	10.48	10.49	10.22	10.76
2018	4.96	4.76	5.16	10.26	9.99	10.53	10.54	10.34	10.75	11.37	11.09	11.65
2019	5.5	5.29	5.72	10.66	10.38	10.93	11	10.79	11.21	11.56	11.28	11.84
2020	6.38	6.15	6.61	11.82	11.54	12.11	11.06	10.86	11.27	12.02	11.74	12.3
2021	6.46	6.24	6.69	12.56	12.27	12.86	12	11.78	12.21	12.71	12.42	13
2022	6.45	6.23	6.68	13.02	12.72	13.31	12.08	11.87	12.29	12.87	12.59	13.16
2023	7.12	6.88	7.35	13.12	12.82	13.42	13.02	12.8	13.24	12.95	12.67	13.24
